# Computational characterization of the structural and mechanical properties of nanoporous titania†

**DOI:** 10.1039/c9ra02298h

**Published:** 2019-05-16

**Authors:** Ziwei Xu, Li Zhang, Lin Wang, Jie Zuo, Mingli Yang

**Affiliations:** Institute of Atomic and Molecular Physics, Sichuan University Chengdu 610065 China lizhang@scu.edu.cn; School of Computer Science, Sichuan University Chengdu 610065 China zuojie@scu.edu.cn; Research Center for Materials Genome Engineering, Sichuan University Chengdu 610065 China

## Abstract

Nanoporous titania is one of the most commonly used biomaterials with good biocompatibility and mechanical strength. Understanding to the influence of pore structures on their performances is crucial for the design and preparation of titania-based materials. Two kinds of structural models for nanoporous titania were constructed and used to investigate the effect of pore size and/or porosity on their mechanical properties by using molecular dynamic simulations with the Matsui–Akaogi potentials. The porous structures were relaxed and their elastic constants were computed and used to evaluated their bulk, shear and Young's moduli. Overlap effect in small pores, pore size and porosity have considerable influence on computed elastic moduli. Compared to bulk rutile TiO_2_, reduced mechanical moduli were predicted. Simulations on uniaxial tensile tests revealed an anisotropic stress–strain relationship and a brittle-to-ductile transition for structures with large porosities. Fracture failure was predicted for all the studied porous structures. The maximum stress decreases with pore size and porosity, while the corresponding strain decreases with pore size, but increases with porosity.

## Abstract 2

Nanoporous titania is one of the most commonly used biomaterials with good biocompatibility and mechanical strength.

## Graphical abstract

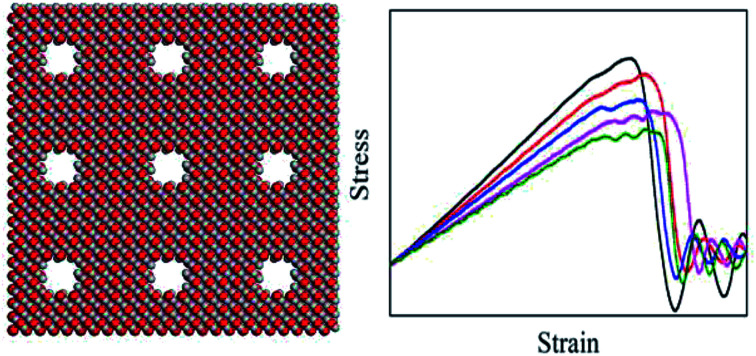

## 1. Introduction

Porous materials can be classified into three types by their pore sizes: microporous (<2 nm), mesoporous (2–50 nm) and macroporous materials (>50 nm). Nanoporous materials are defined as those porous materials with pore diameters of 1–100 nm.^1^ Nanoporous materials have shown great potential in catalysis, hydrogen storage, drug delivery and biological medicine, *etc.*^2–5^ because of their high surface-to-volume ratios, moderate and tunable pore sizes, pore shapes, porosity and framework compositions. Being used as biomedical biomaterials for tissue engineering and drug delivery, some porous materials with good biocompatibility and bioreductivity have been characterized, such as porous titanium and porous calcium phosphates. In fact, implant three-dimensional substitute materials with porous structures are widely used in the treatment of bone trauma in clinics because of their good osteoreductivity that is essential for bone regeneration.^6–9^ The pore structures provide ideal anchors for biomolecules/cell adhesion, proliferation, differentiation and formation of new organs. On the other hand, the porous materials by careful design may possess mechanic strengths that are sufficient to support damaged organs or tissues.^10,11^ Typically, most biological implant materials for bone repair have a Young's modulus higher than those of hard tissues. Stress occlusion can impede bone remodeling and healing, resulting in increased bone porosity. One of the major problems associated with implants in orthopedic surgery is the mismatch in Young's modulus between natural bone and implants. One way to alleviate this problem is to reduce the Young's modulus of the materials by introducing holes,^12–14^ thereby minimizing damage to tissues near the implant and ultimately extending device lifetime. In addition, bone/implant fixation is achieved by the mutual intersection between the bone and the porous implant matrix.^15^

Among various bone graft substitutes from natural and synthetic materials, titanium dioxide (TiO_2_) is considered to be a highly suitable biocompatible material for bone anchoring implants due to its good biocompatibility, osteoconductivity and osteoinductivity.^16–19^ In fact, TiO_2_ exhibits little or no toxicity in both *in vitro* and *in vivo* experiments.^20^ Extensive studies from their preparation, characterization, through applications have been conducted for various TiO_2_-based materials. Fattakhova-Rohlfing *et al.*^21^ surveyed the synthesis of various porous TiO_2_ nanostructures including membranes, spheres, fibers, *etc.* Sabetrasekh *et al.*^22^ reported that porous TiO_2_ particles exhibit better activity in cell proliferation, but less toxicity than commercial bone grafts. Natix®, Straumann® BoneCeramic and Bio-Oss®. Haugen *et al.*^23^ developed a high-interconnectivity and high-porosity TiO_2_ that favors osseous integration, union and regeneration. Santos *et al.*^24^ explored the mechanical properties of biomedical titanium dioxide films prepared by anodizing on commercial pure Ti (cp-Ti), including nanohardness, elastic modulus and brittleness. The modified films have relatively high nano-hardness and small elastic modulus in comparison with the cp-Ti. The reduction in modulus of elasticity was attributed to the porosity and inherent roughness of the oxide surface.

A number of computational studies have been conducted to explore the structures and properties of rutile titania and to help with the design of rutile-based materials. The computations focused mainly on the electronic, thermodynamic and mechanical properties of bulk or nano-sized TiO_2_. For example, Mashreghi^25^ calculated the thermal expansion coefficients of rutile titania nanoparticles under 300–1000 K by means of molecular dynamics (MD) simulations with the Buckingham potentials. Kim *et al.*^26^ reproduced the lattice structures, thermal expansion coefficients and bulk modulus of rutile, brookite and anatase by using molecular dynamics simulations with the Morse interactions. Chung and Buessem^27^ used Voigt–Reuss–Hill (VRH) approximation to calculate the elastic modulus of isotropic polycrystalline TiO_2_ based on the anisotropy single crystal elastic constant. The results are in good agreement with the measured polycrystalline elastic modulus. Mahmood *et al.*^28^ performed a first-principles calculation of rutile TiO_2_ using the general gradient approximation (GGA) proposed by Perdew–Burke–Ernzerhof (PBE), and found that the single crystal elastic constant (*C*_*ij*_), bulk modulus (*B*), Young's modulus (*Y*), shear modulus (*G*), linear bulk modulus (*B*_a_) and the corresponding single crystal elastic constant (*S*_*ij*_) alternately increase or decrease with pressure. However, most of those studies focused on non-porous crystalloid rutile TiO_2_. In the research of mesoporous titania, Solveyra *et al.*^29^ performed molecular dynamics simulations of water confined in mesoporous TiO_2_-rutile pores at different water contents, obtaining water density and diffusion coefficient as a function of distance from the surface. Similarly, Velasco *et al.*^30^ performed a detailed analysis of the hydrodynamics in the pore space of titanium dioxide by a combination of experimental nuclear magnetic resonance NMR data and MD simulation. Gautam *et al.*^31^ used MD simulation to study the structural and kinetic properties of propane restricted in mesoporous TiO_2_.

Compared to tremendous studies on crystallized titanium dioxides, fewer researches have been conducted to their porous samples. Precise design and control over the pore structures is crucial for modulating the properties of porous TiO_2_. Computational studies provide an effective way to establish the correlation between pore structures and performances. In this work we designed various computational models for porous titania with different pore sizes and porosities, and studied their structural and mechanical properties. Our purpose is to figure out the influence of pore structures on the properties of porous titania, which would be helpful for the design and preparation of titania-based materials.

## 2. Methodology

**Fig. 1:**
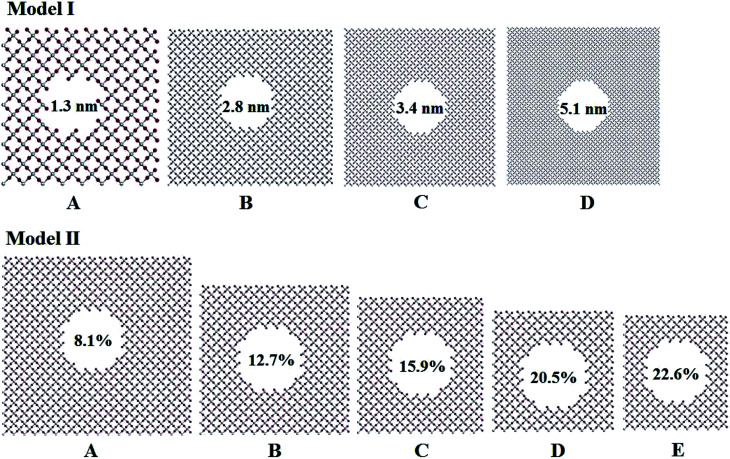
Pore structures with different diameters and porosity in titania.

Titania has three phases in nature, rutile, anatase, and brookite.^32^ Rutile TiO_2_ is thermodynamically stable in its bulk form with a large crystallite size at normal pressure and temperature up to its melting point 1830 K.^33^ Rutile belongs to *P*4_2_/*mnm* space group in which a titanium atom sits at the center of the unit cell and is surrounded by an octahedron formed by six oxygen atoms. The centered Ti atom has six coordinates, while each O atom is coordinated by three Ti atoms. Starting with the rutile TiO_2_ structure, two series of computational models for porous titania were designed and constructed by excavating one cylinder along the *z*-axis direction. In Model I, all of the rutile TiO_2_ porous structures were established at a fixed porosity of 8.1% and the cylinders are 1.3, 2.8, 3.4 and 5.1 nm in diameter, labeled as IA, IB, IC and ID, respectively. This model was used to evaluate the effect of pore size on the structures and properties of porous titania. In Model II, cylinders with a fixed diameter of 2.8 nm were excavated in the supercell. The porosity (from 8.1% to 22.6%) was controlled by changing the number of unit cells in the supercell. This model was used to evaluate the effect of porosity on the structures and properties of porous titania, labeled as IIA, IIB, IIC, IID and IIE, respectively. In both models, the Ti/O ratio is kept to 1 : 2 (ref. 29) and the wall thickness between adjacent pores are larger than 1 nm, as shown in Fig. 1. The three-dimensional structures of Model I and Model II were also given in Fig. S1.† The structural parameters of the constructed models are presented in Table S1.†

Arising from the extensive research interest, several kinds of force fields have been proposed for titanium dioxide.^34–46^ The Matsui–Akaogi (MA) force fields^46^ are commonly used to describe the TiO_2_ systems. Other potentials used for the TiO_2_ systems include modified-MA^42,43^ and MS-Q.^44,45^ Using molecular dynamics simulations with the MA force fields, Matsui and Akaogi^46^ calculated the structure and physical properties of four kinds of TiO_2_. Naicker *et al.*^36^ and Fuertes *et al.*^38^ simulated the surface energy and optical properties of TiO_2_ nanoparticles using MD simulations with the MA potential. The MA potential was also used to simulate the surface structure of TiO_2_ (ref. 35) and the deformation of anatase-type nanocrystals under uniaxial stretching and compression at room temperature.^37^ In addition, the MS-Q potential^44,45^ also produced accurate descriptions for the structures and properties of TiO_2_ crystal.^39–41^ The MA potential is defined as1
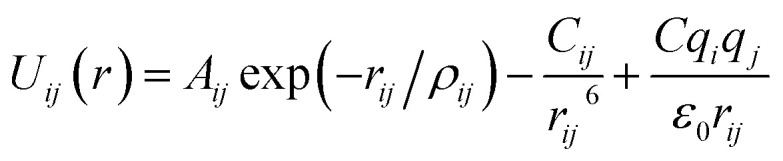
where *U*_*ij*_(*r*), *C*_*ij*_ and *r*_*ij*_ are the interaction energy, van der Waals (vdW) coefficient and distance between atoms *i* and *j*, respectively. *ρ* and *q* are ionic-pair dependent length parameter and atomic charge, respectively. *ε*_0_ is the dielectric constant in vacuum. Alderman and Skinner *et al.*^43^ further proposed a modified-MA potential as2
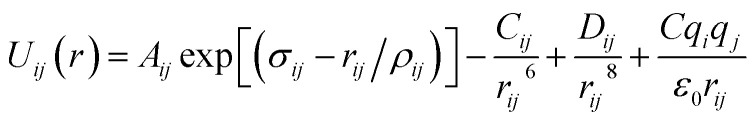
where *σ* is an interaction-dependent length parameter. The MS-Q potential model is defined as3
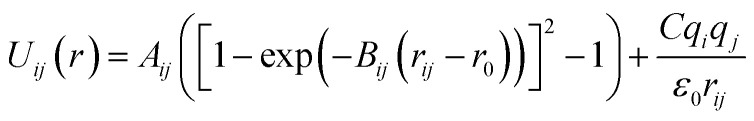
where *A*_*ij*_, *r*_0_, and *B*_*ij*_ are the parameters of Morse potential. *r*_0_ is the equilibrium interatomic separation. The above mentioned three potentials were employed in this work. The parameters are listed in Table S2–S4 in ESI.†

Molecular dynamics simulations were carried out using the LAMMPS code.^47^ The time integral of the Newton's equation of motion was performed using the velocity Verlet algorithm, and the time step was set to 1.0 fs. The Nosé–Hoover thermostat^48,49^ was used to control the temperature. The periodic boundary conditions were applied in the three directions. All the structures were firstly relaxed with the conjugate gradient (CG) algorithm,^50^ and then the constant-pressure constant-temperature (NPT) ensemble was employed for simulations at given temperatures. Particle–particle particle-mesh (PPPM) method was applied to minimize errors in long range terms coulombic. The cut-off of electrostatic interaction and van der Waals interaction used in all the simulation was set to 10 Å. Each simulation was carried out for 1.2 ns. The variations in potential energy with simulation time of all the structures are shown in Fig. S2–S4 in ESI.† All the systems reach their equilibrium at about 200–500 ps. The simulations continue and the data of last 400 ps were used for analysis.

The elastic modulus of rutile TiO_2_ are computed with the elastic constants, which are obtained using the energy-strain theory:4
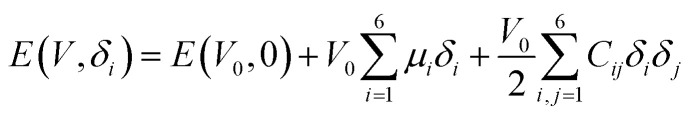
and5
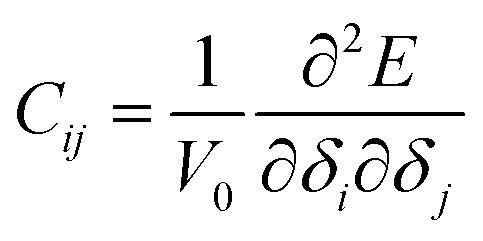
where *V*_0_ and *E*(*V*_0_,0) represent the volume and energy of the unstrained structure. *μ*, *δ* and *C*_*ij*_ denote the stress tensor, strain tensor and elastic constants respectively. Six independent elastic constants, *C*_11_, *C*_12_, *C*_23_, *C*_33_, *C*_44_ and *C*_66_, are defined for the tetragonal bulk and mecroporous rutile TiO_2_ structures.^51^ The maximum strain was set to 1% in the evaluation of elastic constants. Based on the constants, bulk modulus (*K*), shear modulus (*G*) and Young's modulus (*E*) and Poisson ratio (*η*) were then computed with the Voigt–Reuss–Hill method.^52–54^ The calculations of elastic constants were carried out for the equilibrated structures at 300 K. The details of *K*, *G*, *E*, *η* calculations are given in the ESI.† The volume thermal expansion coefficient (*β*) is defined as6
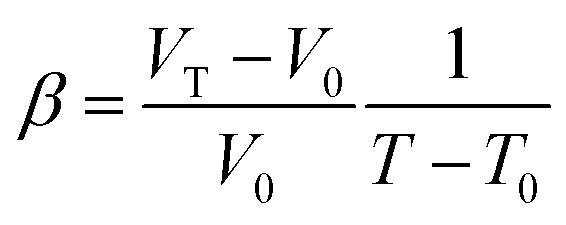
where *V*_0_ and *T*_0_ represent the initial reference volume and temperature. *V*_T_ represents the volume at temperature *T*. The *β* variations of titanium dioxide were explored at temperatures between 300 K and 1000 K with a temperature separation of 100 K.

## 3. Results and discussion

First, we examined the reliability of the potentials by examining the structures and properties of bulk rutile TiO_2_. A 9 × 8 × 18 supercell with 7776 atoms was established. The simulated lattice parameters are given in Table 1, together with the measured and calculated results in previous studies.^55,56^ The three potentials produce comparable results, and the MA and MS-Q results match well with the measurements.^56^

**Table 1:** Lattice parameters of bulk rutile TiO_2_

	*a* (Å)	*b* (Å)	*c* (Å)	*α* (°)	*β* (°)	*γ* (°)
MA	40.515	36.013	54.332	90.00	90.00	90.00
Modified MA	43.722	38.864	54.844	90.00	90.00	90.00
MS-Q	40.155	35.694	52.772	90.00	90.00	90.00
Rao *et al.*^a^	41.346	36.752	53.262	90.00	90.00	90.00
Camargo *et al.*^b^	41.652	37.024	53.748	90.00	90.00	90.00

a Measured in ref. 56.

b First-principle calculations in ref. 55.

The computed six elastic constants of rutile TiO_2_ are given in Table 2. Previous calculations^33,57^ produced similar results with experiments.^58^ Our simulations with the MA and the MS-Q potentials are in good agreement with other studies. The modified-MA results deviate considerably from the measurements, in particular to the shear moduli. The MA potential was thus selected to simulate the elastic properties of porous rutile TiO_2_.

**Table 2:** Elastic constants (in GPa) and moduli (in GPa) of rutile TiO_2_

	*C*_11_	*C*_33_	*C*_12_	*C*_23_	*C*_44_	*C*_66_	*K*	*G*
Calc.^33^	292	471	192	147	114	236	233	113
Calc.^57^	267	483	165	152	122	212	217	128
Expt.^58^	268	484	175	147	124	190	210	113
MA	313	430	220	139	121	220	228	115
Modified-MA	283	392	309	185	143	155	255	1
MS-Q	331	535	186	158	129	211	241	132

**Fig. 2:**
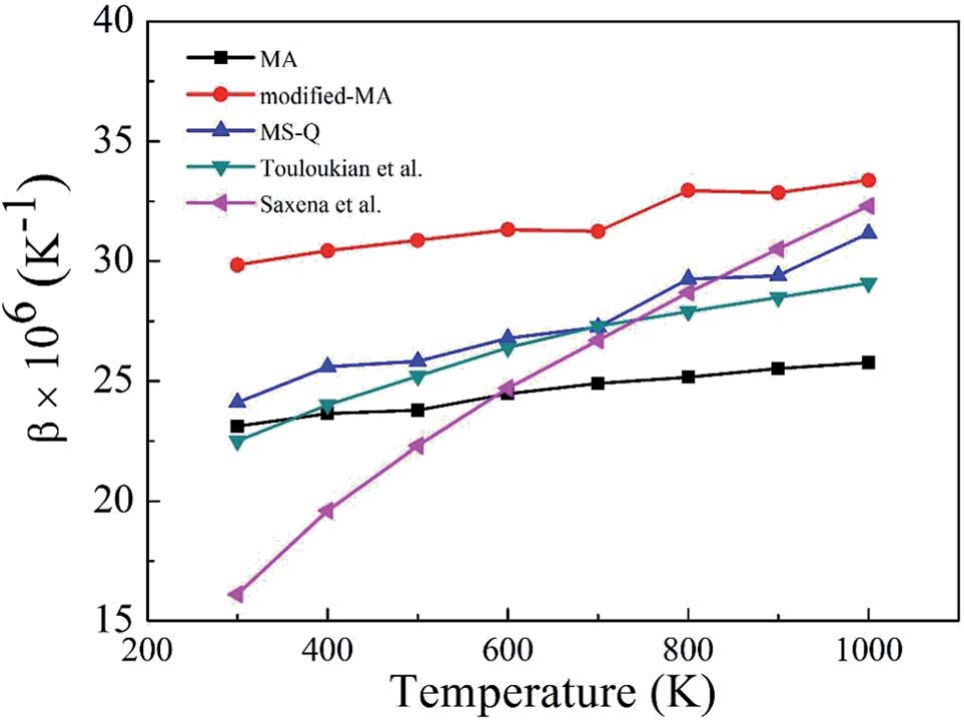
Volume thermal expansion coefficient (*β*) of rutile TiO_2_ at different temperatures. Experimental values are given for comparison.

Fig. 2 depicts the *β* values of bulk rutile TiO_2_ calculated by using the three force fields as a function of temperature. In general, the *β* values increase with temperature, indicating that the volume of rutile TiO_2_ positively expands when temperature increases. Touloukian *et al.*^60^ and Saxena *et al.*^59^ observed similar *β* values at high temperature of 700–1000 K, but distinct values at low temperature of 300–700 K. The three potentials predict a similar trend for the *β* values, which are however different in magnitude. The values by the modified-MA are the largest at the same temperature, followed by the MA and the MS-Q results. Our calculations with the MA and the MS-Q results are close to the measurements of Touloukian *et al.*^60^ The MS-Q potential was thus selected to calculate the volume thermal expansion coefficients of the porous structures.

**Fig. 3:**
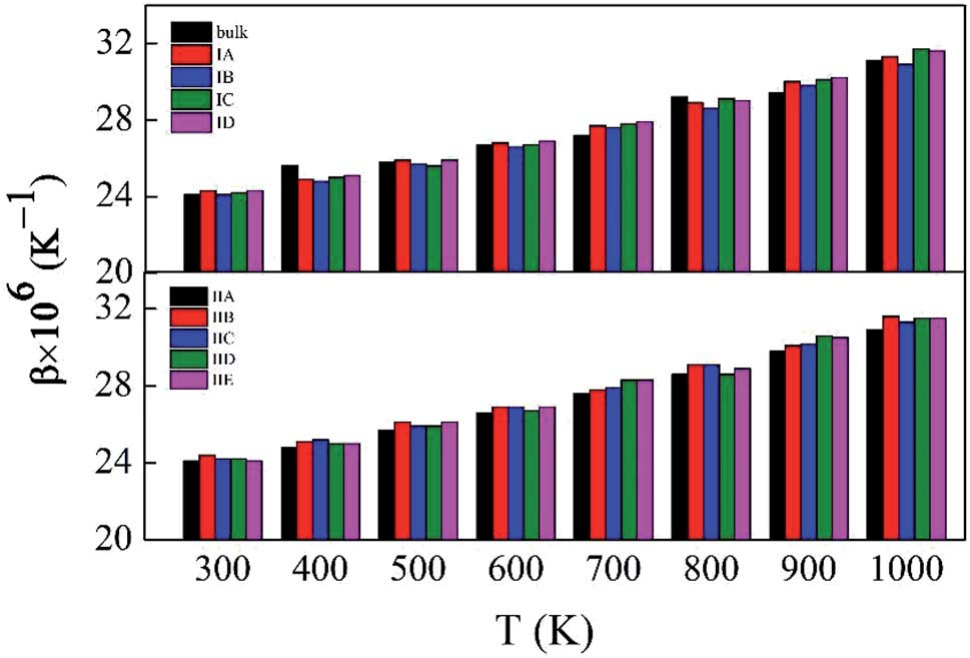
Volume thermal expansion coefficients (*β*) of the porous rutile TiO_2_ structures with different pore sizes (up) and porosities (bottom).

Fig. S5 and S6,† which have similar shapes with Fig. 1, in ESI,† show the pore structures with different pore sizes and porosities in rutile TiO_2_ after relaxation with the MA force field. Compared to Fig. 1, the atoms on the surface of the pores are rearranged to some extent. Both the surface oxygen and titanium atoms basically retain their interatomic connections with only small displacements around their original locations. The locations of inner atoms far away from the holes are almost unchanged. Table 3 lists the structural parameters of the pores in Model I and II. Compared to the unrelaxed structures (Table S1†), in both models the total volumes of the cubic boxes decrease after relaxation under the isobaric–isothermal ensemble. Accordingly, the pore sizes become smaller, but the changes are less than 4%. Atoms have a tendency to move toward the vacuum holes, resulting to the reduction of pore size and the distortion of pore edges. In both models, moreover, the surface areas of the pores and porosity also have a little decrease. Table 3 also presents the specific surface area (*γ*) of all the porous models. *γ* decreases with pore size in Model I, and increases with porosity in Model II. Fig. 3 shows the volume thermal expansion coefficient (*β*) of the porous rutile TiO_2_ structures. In both models, *β* increases with temperature, which is in agreement with many other materials. At some temperatures, *β* increases with pore size and porosity, but, in general, their changes are rather small. Moreover, the *β* values are comparable with that of bulk rutile TiO_2_.

**Table 3:** Structural properties of rutile TiO_2_ pore structures after relaxation using MA force field

Model	Pore size (nm)	Surface area (Å^2^)	Total volume (Å^3^)	Porosity (%)	Specific surface area (×10^−2^ Å^−1^)
**Model I**					
IA	1.2	1384	39 053	8.1	3.5
IB	2.7	2351	149 432	7.9	1.6
IC	3.3	3125	247 644	8.3	1.2
ID	5.0	4449	523 809	8.0	0.8

**Model II**					
IIA	2.7	2351	149 432	7.9	1.6
IIB	2.7	2390	95 796	12.5	2.4
IIC	2.7	2377	76 179	15.7	3.1
IID	2.7	2396	59 139	20.4	4.1
IIE	2.7	2389	53 688	22.4	4.4

Six independent elastic constants, *C*_11_, *C*_12_, *C*_23_, *C*_33_, *C*_44_ and *C*_66_ were computed for the porous structures with the MA force fields, which are given in Table 4. The mechanical stability of these structures was examined using the following conditions:^61,62^*C*_11_ > 0, *C*_33_ > 0, *C*_44_ > 0, *C*_66_ > 0, *C*_11_–*C*_12_ > 0, *C*_11_ + *C*_33_ − 2*C*_23_ > 0, and 2*C*_11_ + *C*_33_ +2*C*_12_ + 4*C*_23_ > 0. These conditions are satisfied by the computed results of all the structures. The results of bulk rutile TiO_2_ are also given for comparison. For Model I, the elastic constants of *R* = 1.3 nm (IA) have rather large changes compared to the corresponding bulk ones. Because of the serious overlap effect of surface atoms in the small pore, IA exhibits different features from the other models. The overlap effect reduces in the models from IB, IC to ID. The computed elastic constants are comparable for IB and IC, and change to some extent for ID. Moreover, changes in *C*_11_, *C*_12_ and *C*_66_ are more remarkable than in *C*_23_, *C*_33_ and *C*_44_. For Model II, the elastic constants decrease with increasing porosity. Similarly, the decrease in *C*_11_, *C*_12_ and *C*_66_ are more remarkable than in *C*_33_, *C*_23_ and *C*_44_. Therefore, both pore size and porosity have influence on the magnitude of elastic constants, especially in *C*_11_, *C*_12_ and *C*_66_.

**Table 4:** Elastic constants (GPa), elastic moduli (GPa) and Poisson's ratio of porous rutile TiO_2_ structures

Model	*C*_11_	*C*_33_	*C*_12_	*C*_23_	*C*_44_	*C*_66_	*K*	*G*	*E*	*η*
Bulk	313	430	220	139	121	220	228	115	295	0.28

**Model I**										
IA	204	382	132	87	96	222	153	87	220	0.26
IB	313	388	220	120	102	220	215	108	277	0.28
IC	310	384	213	117	102	213	211	107	275	0.28
ID	261	388	189	123	104	93	197	84	220	0.31

**Model II**										
IIA	313	388	220	120	102	220	215	108	277	0.28
IIB	287	369	202	118	100	207	202	102	262	0.28
IIC	223	354	160	103	95	184	170	92	235	0.26
IID	167	334	106	78	87	140	129	78	196	0.24
IIE	131	324	79	68	83	116	107	72	176	0.22

**Fig. 4:**
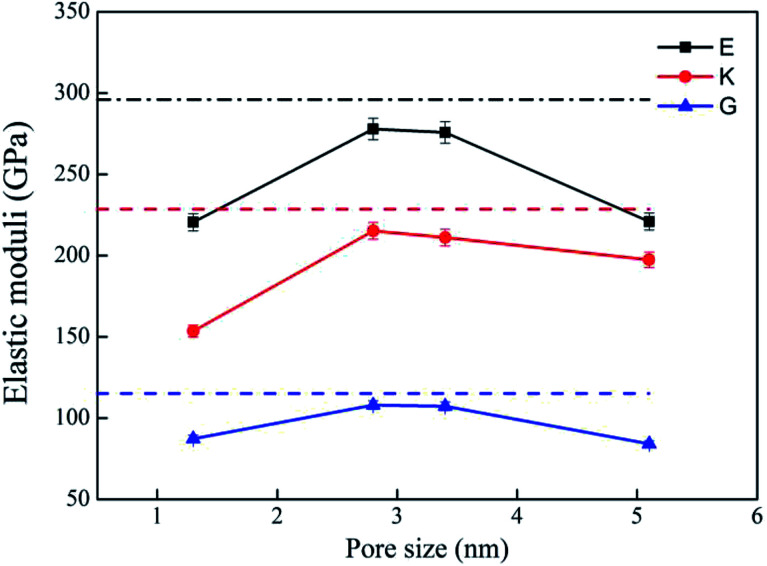
Elastic moduli of porous rutile with different pore sizes. The corresponding values of bulk rutile are presented as dotted line.

**Fig. 5:**
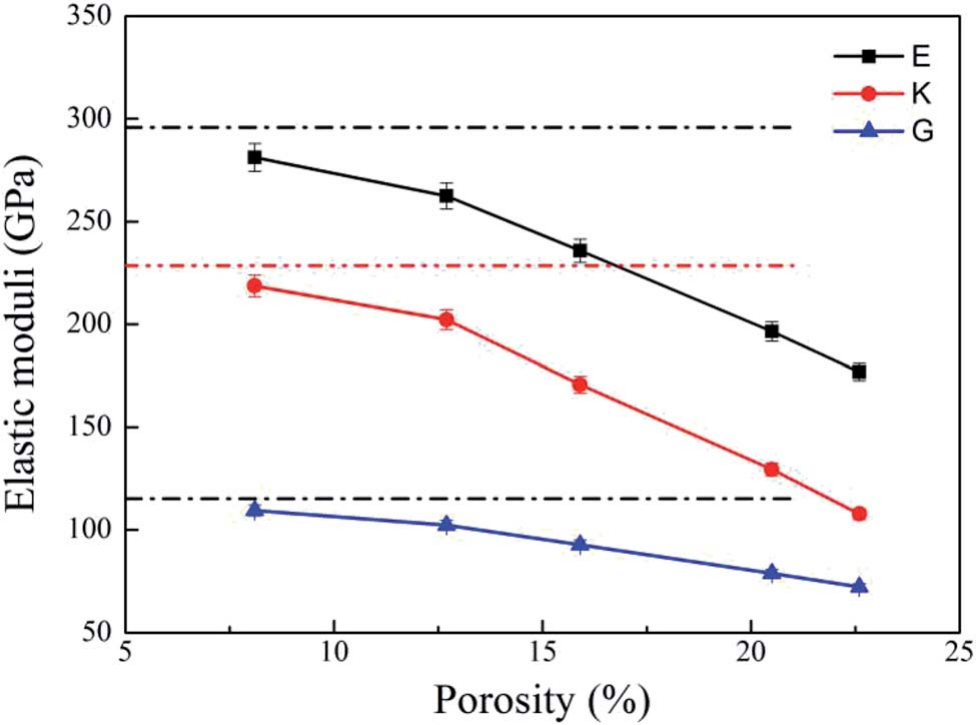
Elastic moduli of porous rutile with different porosities. The corresponding values of bulk rutile are presented as dotted line.

Bulk modulus (*K*) and shear modulus (*G*) are used to measure the rigidity of a material resisting to the volume deformation and shape deformation under external forces. Young's modulus (*E*), a proportion of stress and strain, is customarily used as a measure of rigidity of a crystalline solid. Fig. 4 and 5 display the variation of these moduli with pore diameter and porosity, respectively. The moduli of bulk rutile TiO_2_ are also given in the figures. At the same pore diameter or porosity, an order of *E* > *K* > *G* is always noted. Those three moduli are smaller than the corresponding bulk values. Except IA, the three moduli decrease with pore size and porosity, which again illustrates the overlap effect in small nanopores. The reduced moduli in porous rutile TiO_2_ are in agreement with the observations for the rigidity reduction of other porous materials. For example, Li *et al.*^63^ verified that the elastic moduli of porous nickel decrease as the pore size increases. The reduction was attributed to the increased load and moment on the pore walls. It is interesting to note that the decrease of *E* and *G* with porosity (Fig. 5) is approximately between linear and quadratic functions. Kováčik *et al.*^64,65^ proposed two models of permeability, which are given as

7 *E* = *E*_0_(1 − *P*/*P*_c_)^*f*_*E*_^

8 *G* = *G*_0_(1 − *P*/*P*_c_)^*f*_*G*_^

where *E* and *G* are the effective Young's modulus and shear modulus of porous material, respectively with the porosity of *P*. *E*_0_ and *G*_0_ are the Young's moduli and shear moduli of bulk material. *P*_c_ is the porosity at which *E* or *G* becomes zero. *f*_*E*_ and *f*_*G*_ are the characteristic exponents of the porous samples. In general, the characteristic indices *f*_*E*_ and *f*_*G*_ are 1.10–1.70 and 1.00–1.40, complying with the computed trend shown in Fig. 5.

The ductility/brittleness variations have been observed in other porous materials. Meille *et al.*^66^ reported that porous alumina, which has a fixed pore size, becomes brittler when its porosity increases. The variation was attributed to the thinner walls in the structures with larger porosities, which are more difficult to resist against shear loads. Poisson's ratio (*η*) represents the ratio of transverse strain to axial strain when a material is deformed along the direction of load. It is an elastic constant reflecting transverse deformation of a material. The material with a *η* indicates that the lateral deformation amount is larger than the longitudinal deformation before the plastic deformation occurs. On the contrary, the lateral deformation is smaller than the longitudinal deformation amount. The value of Poisson's ratio (*η*) is often used to estimate the ductility/brittleness with a critical point of 0.25 or lower for a brittle material and 0.33 or higher for a ductile material.^67,68^ Bulk rutile TiO_2_ is brittle, but little is known about the brittleness changes when the pores are introduced. The *η* values are between 0.26 and 0.31 for the structures in Model I and between 0.28 and 0.22 for the structures in Model II. However, one notes that the *η* values were computed by assuming the structures are isotropic, but the porous rutile TiO_2_ are anisotropic. Therefore, the estimation with isotropic *η* values is not feasible for predicting the ductility/brittleness of the porous structures.

**Fig. 6:**
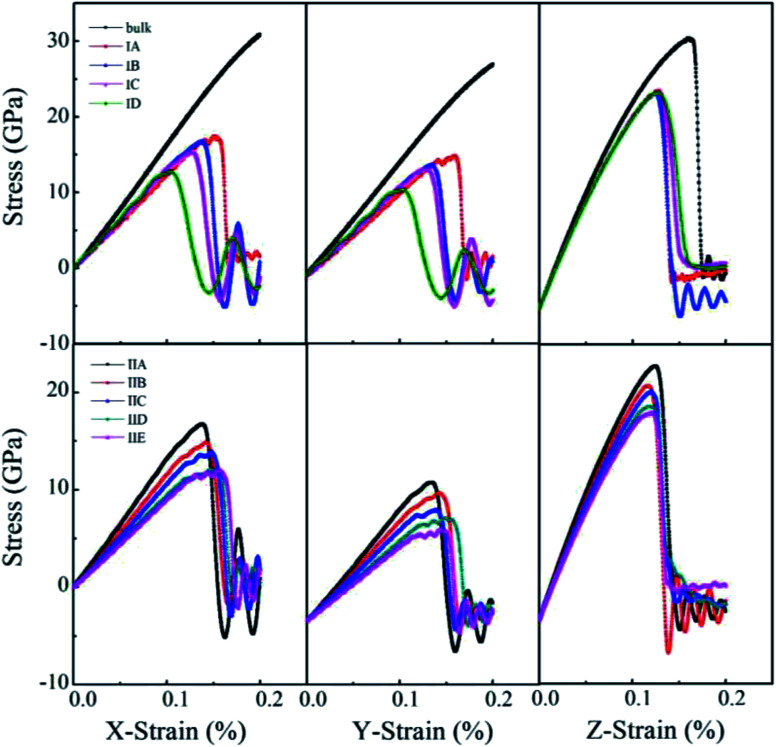
Stress–strain curves of porous rutile with different pore sizes (top) and different porosities (bottom).

Uniaxial tensile measures the mechanical responses of a material subjected to a uniaxial load in an external environment. Molecular dynamics simulations have been conducted by many authors^69–72^ to investigate the elastic limit, tensile strength, yield point, yield strength and other tensile properties of crystals, polymers and ceramics. The stress–strain behavior of porous rutile TiO_2_ was simulated in this work by performing uniaxial tensile tests on the model structures. The simulations were carried out on the *x*, *y* and *z* directions of rutile TiO_2_, which are perpendicular to the (001), (100) and (010) facets, respectively. In each stress–strain relationship, only one direction is stretched, while pressure in the other two vertical directions was set to zero. The strain rate of stretching was set to 1.0 × 10^10^ s^−1^ and temperature was kept at 298 K. The stress–strain curves were obtained for all the structures in Model I and Model II, as shown in Fig. 6. The structures were stretched from 0 to 20% under an external load, and the corresponding stresses are *σ*_*xx*,_*σ*_*yy*_ and *σ*_*zz*_ in the three directions, respectively. The fluctuations in the two directions perpendicular to the tensile direction are rather small and are not presented.

For the structures in Model I, their stress–strain curves follow the same behaviors: elastic at small strains, and then failure at great strains. However, the curves vary with pore size and are different from those of bulk rutile TiO_2_. The porous structures have smaller elastic moduli than the bulk and their failure points are smaller than those of the bulk in the three directions. In the *x* and *y* directions, the structure with a large pore has a slightly smaller elastic modulus than the structure with a small pore. The maximum stresses *σ*_*xx*_ of Model IA–ID in the *x* direction are 17.4, 16.5, 15.4 and 12.7 GPa, the strains corresponding to the maximum stresses are 15.2, 13.6, 12.5 and 10.4%, respectively. In the *y* direction, the maximum stresses are 17.5, 16.2, 15.7 and 12.6 GPa and their corresponding strains are 16.0, 13.5, 12.9 and 10.3%, respectively. The maximum stresses *σ*_*xx*_ and *σ*_*yy*_, as well as their corresponding strains decrease with pore size, indicating that the structures with larger pore sizes at the same porosity have lower tensile strengths. Therefore, the structure with a large pore tends to be fractured at a small load in the *x* and *y* directions. In the *z* direction, the maximum *σ*_*zz*_ values are larger those in the other two directions and have few changes with pore size. Their corresponding strains also have rather small changes, 12.7%, 12.5%, 12.7% and 12.6%, respectively. In the direction parallel to the cylindrical pore, therefore, the mechanical behaviors have smaller changes than the other two directions.

One notes that the curves for Model IA in the *x* and *y* directions fluctuate when the loads are close to the maximum stresses, which indicates a plastic deformation at a strong stress. Such plastic deformation was not noted in the other three structures in Mode I. IA has the smallest pore size and thinnest wall among the four structures when their porosity is fixed. The thin wall favors a plastic deformation for the rutile TiO_2_. However, the plastic deformation changes into a brittle fracture when the strain increases further. Therefore, the stretching failure in all the structures in Model I is characterized by their brittle fracture, a sudden drop in stress at a given strain.

For the structures in Model II whose pore sizes are fixed and porosities are varying and larger than those of the structures in Model I, their stress–strain curves reflect elastic responses at small strains. However, the curves deviate from the elastic responses at larger strains. The stresses fluctuate with strain, which represents a plastic deformation at this stage. The deformation is more remarkable for the structures with large porosities, resulting from their thinner wall thickness. Brittle fractures occur when the stains increase further. Over the studied stain range, elastic and inelastic, the moduli of the porous structures are smaller than those of the bulk. The stress–strain relationship varies with porosity. In the *x* direction, the maximum stress reaches 16.73, 14.87, 13.9, 12.09 and 12.02 GPa at a strain of 13.68%, 14.2%, 14.78%, 14.58% and 15.54%, respectively. The maximum stress decreases with porosity. The structure with a larger porosity has a lower maximum stress, which, however, corresponds to a larger strain. This is in contrast to the variations found in Model I. The structures in Model II have larger porosities than those on Model I. When the external load is larger than 10%, the stress varies in an inelastic pattern with the strain. The deviation from elastic response is more remarkable for the structures with larger porosities. The five structures are in the order of IIA > IIB > IIC > IID > IIE. The brittle-to-ductile transition results from the rearrangement of atoms around the pores, leading to a small stress/stain over a certain range of strains. Therefore, IIE has the smallest maximum stress but the largest failure strain. Similar variations were noted in the stress–strain curves in the *y* direction, the maximum stress and the failure strain vary with porosity, but their maximum stresses are lower than those in the *x* direction. The brittle-to-ductile transition was also noted. In the *z* direction, the variations with porosity become less remarkable. The five structures have almost the same stress–strain relationship at small strains. Their maximum stresses are higher than those in the other two directions, and are slightly higher for the structures with larger porosities. The maximum stresses correspond to almost the same strain values. The brittle-to-ductile transition is not clear in this direction, but brittle fractures are noted for all the five structures.

## 4. Conclusion

Two structural models were constructed to mimic the structures of porous rutile TiO_2_. In the first model, the four structures have different pore sizes of 1.3, 2.8, 3.4 and 5.1 nm in diameter, respectively, but have the same porosity. In the second model, the five structures have different porosities from 8.1%, 12.7%, 15.9%, 20.5% and 22.6%, respectively, but have the same pore size. The influence of pore size and porosity on the structures and mechanical properties of porous rutile TiO_2_ were then investigated using MD simulations. The MA potential was selected to predict the structures and elastic properties, and the MS-Q potential was used for computing the volume expansion coefficients.

In the relaxed porous structures, the surface atoms basically retain their interatomic connections with only small displacements around their original locations. The locations of inner atoms far away from the holes are almost unchanged. Their volume thermal expansion coefficients are comparable with that of bulk rutile TiO_2_, and increase with pore size and porosity at the same temperature, but the increments are rather small. The computed elastic constants vary with pore size and porosity, especially for *C*_11_, *C*_12_ and *C*_66_. The structure with the smallest pore size of 1.3 nm exhibit exceptional variations in its elastic constants because of the overlap effect in its small pore. The elastic modulus (*E*, *K* and *G*) were evaluated based on the computed elastic constants. The three moduli are smaller than the corresponding bulk values, decreasing with pore size and porosity.

The uniaxial tensile tests were performed for all the structures in the two porous models with MD simulations. The stress–strain curves were obtained in three directions. The stress varies in an anisotropic way. In the *z* direction parallel to the cylindrical pore the stress behaves close to that of the bulk. In the other two directions, the maximum stress decreases with pore size and porosity. At small strains, elastic response was noted for all the structures. When the strain increases, inelastic response was noted for the structures with large porosities. The deviation from elastic response increases with porosity, and brittle-to-ductile transition was noted. When the strain increases further, brittle fracture where a sudden drop in stress occurs was noted for all the structures. The brittle failure occurs at smaller strains for the structures with larger pores in Model I, but at larger strains for the structures with large porosities, resulting from the brittle-to-ductile transitions. Our simulations show that the mechanical behaviors of porous structures depend closely with pore structures, including their size, density, *etc.* Pore shape and their connectivity may also have great influence on their mechanical properties, which will be investigated in our future work.

## Conflicts of interest

There are no conflicts to declare.

## Supplementary Material

RA-009-C9RA02298H-s001
