# Supplementary material for: Computational characterization of the structural and mechanical properties of nanoporous titania
Source: RSC Adv. 2019 May 16;9(27):15298–306. doi: 10.1039/c9ra02298h (PMC9064309; doi:10.1039/c9ra02298h)
Supplement: RA-009-C9RA02298H-s001 [file RA-009-C9RA02298H-s001.pdf]

Electronic Supplementary Material (ESI) for RSC Advances.
This journal is © The Royal Society of Chemistry 2019

Supplementary Material

Computational characterization of the structural and mechanical properties of nanoporous titania

Ziwei Xu[a], Li Zhang[a,*], Lin Wang[a], Jie Zuo[b,**] and Mingli Yang[c]

[a] Institute of Atomic and Molecular Physics, Sichuan University, Chengdu 610065, China

[b] School of Computer Science, Sichuan University, Chengdu 610065, China

[c] Research Center for Materials Genome Engineering, Sichuan University, Chengdu 610065, China

*Corresponding author. Institute of Atomic and Molecular Physics, Sichuan University, Chengdu 610065, China

** Corresponding author.

E-mall addresses: lizhang@scu.edu.cn (L. Z.), zuojie@scu.edu.cn (J. Z.).

S1. Three-dimensional structures

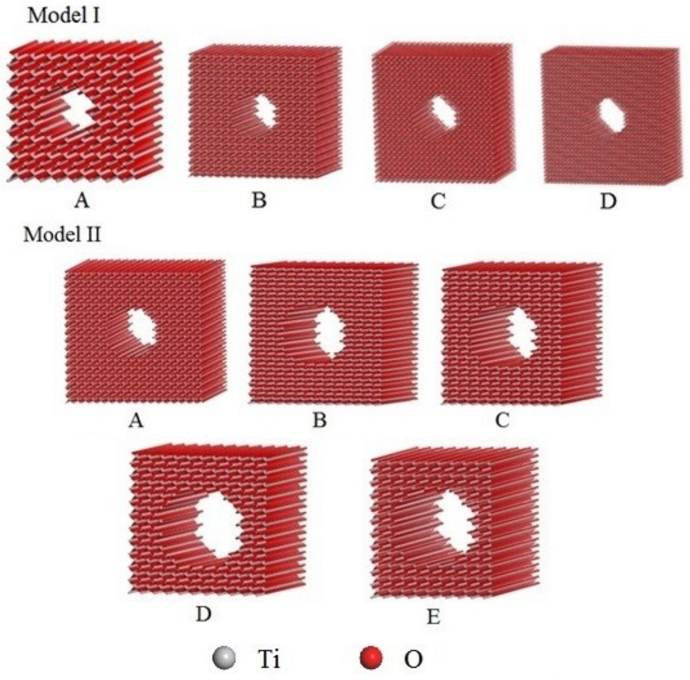

Fig. S1 The three-dimensional structures of Model I and Model II. Gray and red represent Ti and O atom, respectively.

S2. Potential energy of structures

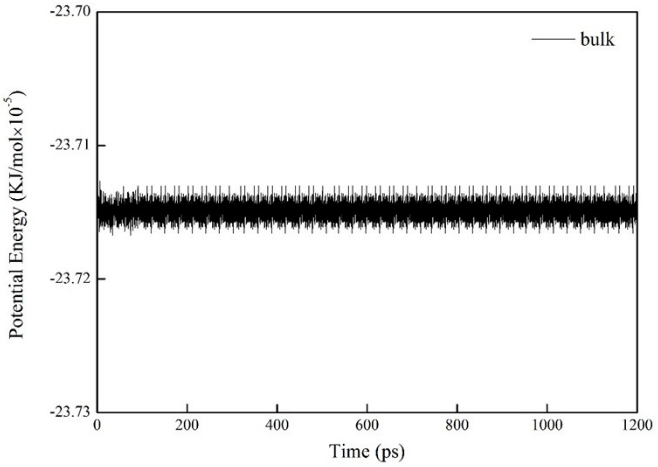

Fig. S2 Potential energy of bulk rutile TiO_2 at 300 K.

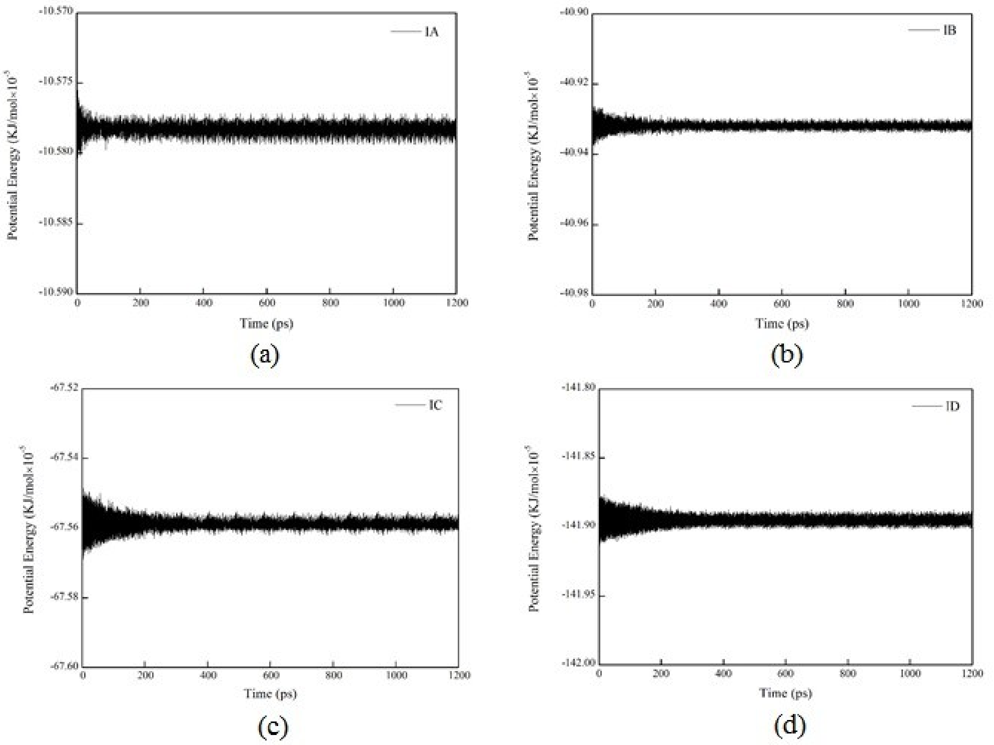

Fig. S3 Potential energy of Model I at 300 K. (a) IA. (b) IB. (c) IC. (d) ID.

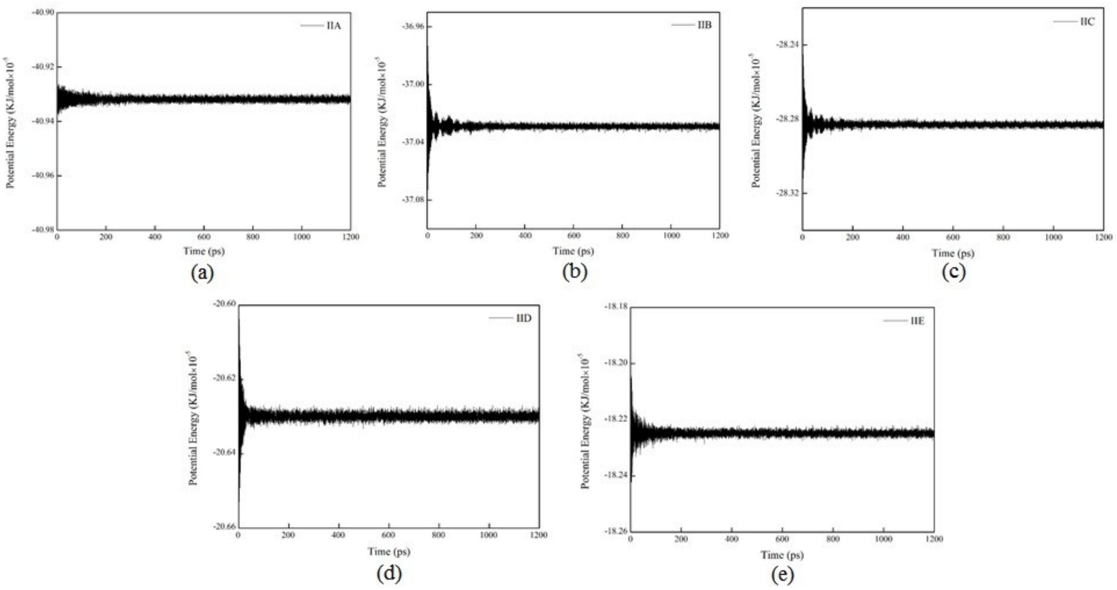

Fig. S4 Potential energy of Model II at 300 K. (a) IIA. (b) IIB. (C) IIC. (d) IID. (e) IIE.

S2. Relaxed Structures

The relaxed porous structures are shown as Fig. S5 and Fig. S6.

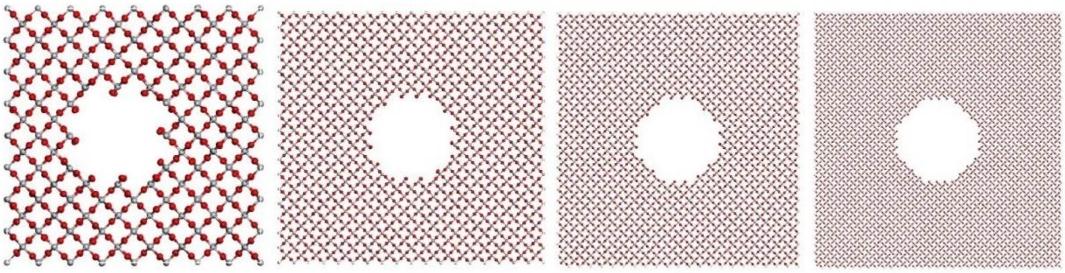

Fig. S5 Relaxed structures of porous titania with different pore sizes.

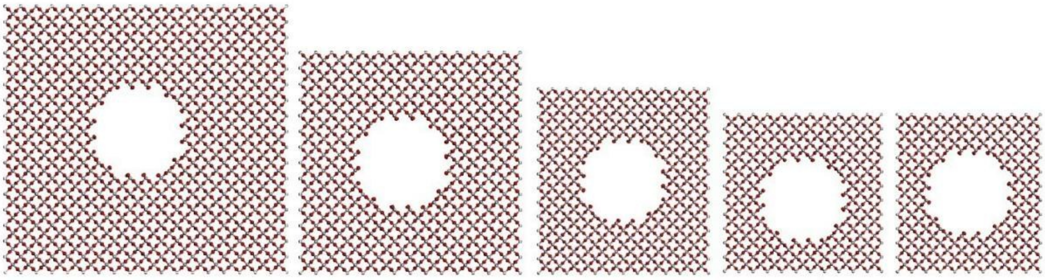

Fig. S6 Relaxed structures of porous titania with different porosities.

S2. Structural parameters

The structural parameters (pore size, surface area total volume, porosity and specific surface area) of the constructed models are listed in Table S1.

Table S1 Structural parameters of the constructed models.

Model	Pore size (nm)	Surface Area (Å^2)	Total volume (Å^3)	Porosity (%)	Specific surface area (×10^{-2} Å^{-1})
			Model I		
IA	1.3	1444	39967	8.3	3.6
IB	2.8	2363	152875	8.1	1.5
IC	3.4	3072	252794	8.3	1.2
ID	5.1	4395	527570	8.0	0.8
			Model II		
IIA	2.8	2363	152875	8.1	1.5

IIB	2.8	2432	97920	12.7	2.4
IIC	2.8	2423	77936	15.9	3.0
IID	2.8	2406	60450	20.5	4.0
IIE	2.8	2399	54955	22.6	4.4

S3. Parameters of three force fields

Table S2. Parameters of the Matsui-Akaogi (MA) force fields

i-j	A_{ij} (kcal/mol)	ρ_{ij} (Å)	C_{ij} (kcal/mol Å^6)
Ti-Ti	717895.18	0.154	121.037
Ti-O	391184.85	0.194	290.489
O-O	271810.46	0.234	697.175

Atomic charges: q(Ti) = 2.196 (e), q(O) = -1.098 (e)

Table S3. Parameters of the modified-MA force fields

i-j	A_{ij} (kcal/mol)	ρ_{ij} (Å)	σ_{ij} (Å)	C_{ij} (kcal/mol Å^6)	D_{ij} (kcal/mol Å^8)
Ti-Ti	415086.9482	0.25	0	18448.30881	11530.193
Ti-O	68339.20028	0.248213237	0	334.6753822	63.41606153
O-O	51053.61919	0.343644974	0	4440.969138	2444.400917

Atomic charges: q(Ti) = 2.196 (e), q(O) = -1.098 (e)

Table S4. Parameters of the MS-Q force fields

i-j	A_{ij} (kcal/mol)	B_{ij} (Å^{-1})	r_0 (Å)
Ti-Ti	0.130784443	1.5543	4.18784
Ti-O	23.70490766	3.640737	1.88265
O-O	0.971234278	1.1861	3.70366

Atomic charges: q(Ti) = 1.151 (e), q(O) = -0.576 (e)

S4. Elastic property

Bulk modulus (*K*), shear modulus (*G*) and Young's modulus (*E*) and Poisson ratio (*η*) were evaluated from the computed elastic constants with the Voigt-Reuss-Hill method:

$$K = \frac{1}{2}(K_V + K_R) \quad \text{(S1)}$$

$$G = \frac{1}{2}(G_V + G_R) \quad \text{(S2)}$$

where the subscript of V and R respectively represent the elastic moduli of Voigt theory and Reuss theory.

$$E = \frac{9KG}{3K + G} \tag{S3}$$

$$\eta = \frac{3K - 2G}{2(3K + G)} \tag{S4}$$

$$K_V = \frac{1}{9}[C_{11} + C_{22} + C_{33} + 2(C_{12} + C_{13} + C_{23})] \tag{S5}$$

$$G_V = \frac{1}{15}[C_{11} + C_{12} + C_{33} + 3(C_{44} + C_{55} + C_{66}) - (C_{12} + C_{13} + C_{23})] \tag{S6}$$

$$\begin{aligned} K_R = [&C_{11}(C_{22} + C_{33} - 2C_{23}) + C_{22}(C_{33} - 2C_{13}) - 2C_{33}C_{12} \\ &+ C_{12}(2C_{23} - C_{12}) + C_{13}(2C_{12} - C_{13}) + C_{23}(2C_{13} - C_{23})]^{-1} \end{aligned} \tag{S7}$$

$$\begin{aligned} G_R = 15\{&4[C_{11}(C_{22} + C_{33} + C_{23}) + C_{22}(C_{33} + C_{13}) + C_{33}C_{12} \\ &- C_{12}(C_{23} + C_{12}) - C_{13}(C_{12} + C_{13}) - C_{23}(C_{13} + C_{23})] / C_X \\ &+ 3(\frac{1}{C_{44}} + \frac{1}{C_{55}} + \frac{1}{C_{66}})\}^{-1} \end{aligned} \tag{S8}$$

$$\begin{aligned} C_X = &C_{13}(C_{12}C_{23} - C_{13}C_{22}) + C_{23}(C_{12}C_{13} - C_{11}C_{23}) \\ &+ C_{33}(C_{11}C_{22} - C_{12}C_{12}) \end{aligned} \tag{S9}$$
